# Gemcitabine plus vinorelbine in elderly or unfit patients with non-small cell lung cancer

**DOI:** 10.1054/bjoc.2000.1304

**Published:** 2000-08-16

**Authors:** G D Beretta, G Michetti, M O Belometti, G Gritti, A Quadri, P Poletti, R Labianca

**Affiliations:** Medical Oncology Unit, Pneumology Unit, Radiotherapy Unit, Ospedali Riuniti, Largo Barozzi 1, Bergamo, 24100, Italy

**Keywords:** elderly patients, unfit patients, NSCLC, gemcitabine, vinorelbine

## Abstract

Cisplatin-based combinations are efficacious in increasing the overall survival of patients with non-small cell lung cancer (NSCLC), but their toxicity makes them unsuitable for elderly and unfit patients. The primary objective of this non-randomized phase II study was to evaluate the feasibility and activity of the gemcitabine plus vinorelbine combination in previously untreated elderly and/or unfit patients with measurable stage III or IV NSCLC. Forty-three patients aged ≥ 65 years or with contraindications against cisplatin treatment (36 males and seven females: median age 66 years; range 48–75: PS 0 = 11, PS 1 = 19, PS 2 = 13) received intravenous (i.v.) gemcitabine 1000 mg m^–2^, followed by vinorelbine 25 mg m^–2^i.v. on day 1 and 8 every 21 days. Fifteen patients (34.9%) achieved partial remission (confidence interval: 27.6–42.2%) for a median duration of 6 months; the median survival of these patients has not yet been reached. A further 15 had stable disease for a median of 4 months and a median survival of 7 months. The 10 patients (23.2%) who experienced disease progression had a median survival of 4 months. Three patients are not evaluable. The 1-year actuarial survival rate is 31.1%. The treatment was well tolerated: only 35% of the patients had grade 3 or 4 granulocytopenia on day 14, none experienced episodes of neutropenic fever, and there was no evidence of severe haematological toxicity upon recycling. Only 9% of the patients suffered from gastrointestinal toxicity (grade 3); increased but reversible transaminase levels were observed in 11.6%. In conclusion, the results of this phase II study show that the combination of gemcitabine and vinorelbine is active and well tolerated in NSCLC, and thus encourage its use in elderly or unfit patients. © 2000 Cancer Research Campaign


British Journal of Cancer (2000) 83(5), 573–576
© 2000 Cancer Research Campaign
doi: 10.1054/ bjoc.2000.1304, available online at http://www.idealibrary.com on IDEAL®

### Gemcitabine plus vinorelbine in elderly or unfit patients with non-small cell lung cancer

**GD Beretta[1], G Michetti[2], MO Belometti[2], G Gritti[3], A Quadri[1], P Poletti[1] and R Labianca[1]**

[1]Medical Oncology Unit, [2]Pneumology Unit, [3]Radiotherapy Unit, Ospedali Riuniti, Largo Barozzi 1, 24100 Bergamo, Italy

**Summary** Cisplatin-based combinations are efficacious in increasing the overall survival of patients with non-small cell lung cancer (NSCLC), but their toxicity makes them unsuitable for elderly and unfit patients. The primary objective of this non-randomized phase II study was to evaluate the feasibility and activity of the gemcitabine plus vinorelbine combination in previously untreated elderly and/or unfit patients with measurable stage III or IV NSCLC. Forty-three patients aged ≥ 65 years or with contraindications against cisplatin treatment (36 males and seven females: median age 66 years; range 48–75: PS 0 = 11, PS 1 = 19, PS 2 = 13) received intravenous (i.v.) gemcitabine 1000 mg $m^{-2}$, followed by vinorelbine 25 mg $m^{-2}$ i.v. on day 1 and 8 every 21 days. Fifteen patients (34.9%) achieved partial remission (confidence interval: 27.6–42.2%) for a median duration of 6 months; the median survival of these patients has not yet been reached. A further 15 had stable disease for a median of 4 months and a median survival of 7 months. The 10 patients (23.2%) who experienced disease progression had a median survival of 4 months. Three patients are not evaluable. The 1-year actuarial survival rate is 31.1%. The treatment was well tolerated: only 35% of the patients had grade 3 or 4 granulocytopenia on day 14, none experienced episodes of neutropenic fever, and there was no evidence of severe haematological toxicity upon recycling. Only 9% of the patients suffered from gastrointestinal toxicity (grade 3); increased but reversible transaminase levels were observed in 11.6%. In conclusion, the results of this phase II study show that the combination of gemcitabine and vinorelbine is active and well tolerated in NSCLC, and thus encourage its use in elderly or unfit patients.
© 2000 Cancer Research Campaign

**Keywords:** elderly patients; unfit patients; NSCLC; gemcitabine; vinorelbine

Lung tumours are the leading cause of cancer deaths, accounting for 150 000 deaths/year in the USA (Parker et al, 1996) and 30 000 deaths/year in Italy (Zanetti et al, 1998). Approximately 80% of these tumours are non-small cell lung cancers (NSCLC), most of which are diagnosed in an advanced phase and are thus not suitable for local–regional treatment; overall survival at 5 years is only 10–15% (Bunn, 1993).

In the case of tumours that have spread beyond surgically treatable limits (locally advanced and/or metastastatic disease), cisplatin-containing chemotherapeutic regimens have recently been found to be effective not only in terms of response, but also in terms of an, albeit limited, increase in overall survival (Souquet et al, 1993; NSCLCCG, 1995). However, a large number of subjects are not even considered for this treatment because the toxic nature of cisplatin means that it is contraindicated in patients with heart or kidney diseases, as well as in those whose general physical condition is poor.

As a result, new drugs with demonstrated activity against NSCLC, such as the taxanes (Gatzemeier et al, 1995; Fossella et al, 1995), topoisomerase inhibitors (Fukuoka et al, 1992), gemcitabine (Noble and Goa, 1997) and vinorelbine (Lilenbaum and Grece, 1993; Goss et al, 1997) have aroused considerable interest. In particular, vinorelbine has been shown to increase survival in patients aged more than 70 years in comparison with the best supportive care alone (ELCVISG, 1999), and the favourable toxicological profile of gemcitabine allows its use in both unfit and elderly patients.

When administered alone, both gemcitabine and vinorelbine have been found to be effective in approximately 20% of the studied patients (including cisplatin-resistant cases). There are few data concerning their combined use, although the most tolerated schedule seems to be the administration of gemcitabine 1000 mg $m^{-2}$ and vinorelbine 25 mg $m^{-2}$ on day 1 and 8 every 21 days.

The activity of the drugs and the tolerability of this combination prompted us to test it in patients with NSCLC. We first used it in patients who had previously tolerated a cisplatin-containing regimen well and observed a response rate of approximately 13% (with 50% of the patients reaching stable disease). This encouraging result led us to extend its use to chemotherapy-naive patients aged more than 65 years and those whose compromised performance status or concomitant diseases contraindicated the administration of cisplatin.

The primary objectives of this non-randomized phase II study were to determine the efficacy and toxicity of the combination in elderly or unfit chemotherapy-naive NSCLC patients.

### MATERIALS AND METHODS

### Patients

Since March 1997, we have used the gemcitabine plus vinorelbine combination to treat 43 previously untreated stage III (24) or stage IV (19) NSCLC patients (36 males and seven females, with a median age of 66 years: range 48–75), who were considered ineligible for cisplatin treatment on the ground of their age or the

*Received 9 September 1999*
*Revised 2 March 2000*
*Accepted 13 April 2000*

*Correspondence to*: GD Beretta

**Table 1** Patient characteristics

| No. of patients | 43 |
|---|---|
| Age (years) | |
| median | 66 |
| range | 48–75 |
| Sex (M/F) | 36/7 |
| Performance status (ECOG) | |
| 0 | 11 |
| 1 | 19 |
| 2 | 13 |
| Stage | |
| III A | 4 |
| III B | 20 |
| IV | 19 |
| Histological type | |
| Adenocarcinoma | 17 |
| Epidermoid | 11 |
| NSCLC NAS | 12 |
| Large cells | 1 |
| Bronchiolo-alveolar | 1 |
| Mixed | 1 |
| Metastatic sites | |
| Brain | 3 |
| Liver | 6 |
| Adrenal gland | 4 |
| Bone | 6 |
| Lung | 6 |

**Table 2** Physical condition of the patients

| Age | Patients | Performance status (ECOG) | | | Comorbidity |
|---|---|---|---|---|---|
| | (*n*) | 0 | 1 | 2 | |
| > 65 | 26 | 11 | 15 | 0 | – |
| < 65 | 13 | 0 | 0 | 13 | – |
| < 65 | 4 | 0 | 4 | 0 | 2 with nephropathy<br>2 with cardiopathy |

presence of other contraindications (Table 1). Of the 17 patients aged less than 65 years, 13 had an ECOG performance status of ≥2, and four concomitant kidney or heart disease (Table 2).

The four stage IIIa patients included in the study were more than 65 years old, presented contraindications against surgery, and their respiratory function made them ineligible for radiotherapy.

### Treatment regimen

The chemotherapeutic regimen consisted of one intravenous dose of gemcitabine 1000 mg $m^{-2}$ in 100 ml of normal saline administered over 30 min, followed by an i.v. vinorelbine bolus of 25 mg $m^{-2}$ administered over 2 min. 250 ml of normal saline were subsequently administered over 15 min in order to wash out the vein. The treatment was given on days 1 and 8, and repeated every 3 weeks.

The anti-emetic treatment was ondansetron 8 mg in 100 cc of normal saline administered 15 min before the chemotherapy, with a further 4 mg being given per os in the evening of the same day. The patients subsequently received metoclopramide 10 mg i.m. as needed.

Blood counts were performed on days 7, 14 and 20. Liver and kidney function was checked on day 20. In the case of an incomplete recovery of haematological values by day 20, the therapy was delayed for 1 week. The planned dose was reduced by 25% only in the case an incomplete haematological recovery on day 27 or grade 4 toxicity.

### Statistical considerations

In accordance with the optimal two-stage phase II design, the treatment programme was designed to reject a response rate of less than 20% (p0) and provide a statistical power of 80% in assessing the activity of the regimen (in terms of response rate) as 40% (p1) (p1 – p0 = 20%) with an alpha error of less than 0.05 (Simon, 1989).

Forty-three patients were therefore required: 13 for the first and 30 for the second step.

### RESULTS

Of the 43 patients originally included in the trial, three failed to complete the first treatment cycle (one died early, one developed pulmonary cavitation with superinfection, and one refused to continue therapy in our Centre) but were nevertheless included in the response and toxicity evaluations. The median follow-up was 11 months (range: 4–21).

All of the evaluable patients completed at least two treatment cycles, with a median of four cycles per patient (range 0.5–6) for a total of 146 cycles; their measurable lesions were evaluated instrumentally.

Fifteen patients (34.9%) achieved partial remission (95% confidence interval: 27.6–42.2%), with a median response duration of 6 months; the median survival of these patients has not yet been reached. A further 15 had stable disease for a median of 4 months and a median survival of 7 months. The ten patients (23.2%) who experienced disease progression had a median survival of 4 months.

The survival curves (Figure 1 and 2) show an advantage for stage III over stage IV patients, and for responders in comparison with those who progressed; the survival of the patients with stable disease overlapped that of the patients as a whole.

Ten of the patients with stage IIIb disease received radiotherapy after completing the chemotherapy programme; the two who responded well to both chemo- and radiotherapy also underwent complete surgical resection, the histological results of which revealed the presence of active adenocarcinomas.

The treatment was well tolerated: only 35% of the patients experienced episodes of grade 3 or 4 granulocytopenia, all of which occurred occurred on day 14 without any symptoms or fever. There was no need for antibiotics and hospital admission. There was no evidence of any severe haematological toxicity at the time of recycling. Grade 3 thrombocytopenia was observed in 9% of the patients but, once again, only at the nadir.

Gastrointestinal tolerance was excellent, with only 9% of the patients developing short-lived grade 3 toxicity (nausea and vomiting). Increased transaminase levels (less than double normal values) were observed in 11.6% of the patients, but were always reversible.

The excellent tolerability of this combination enabled us to administer an average of 93% of the planned dose (99.2% excluding the inevaluable patients) at a mean dose intensity of 92%.

© 2000 Cancer Research Campaign

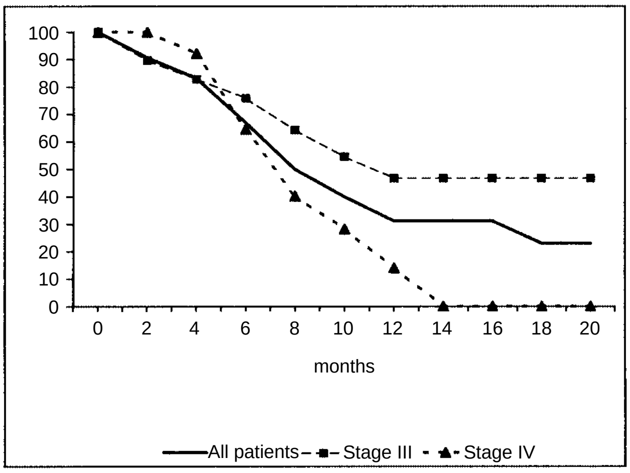

**Figure 1** Overall survival by stage

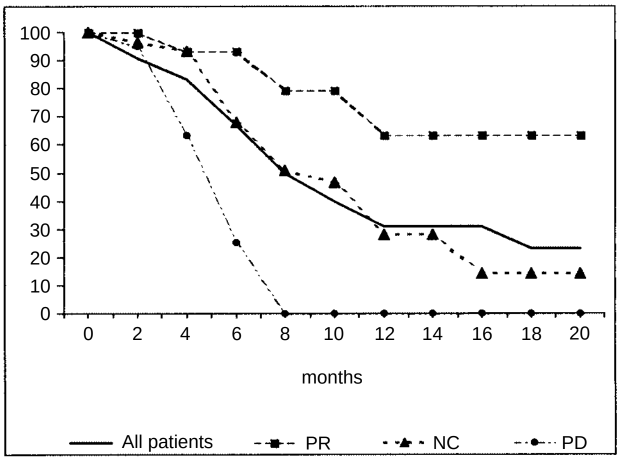

**Figure 2** Overall survival by response

### DISCUSSION

The ASCO guidelines for the treatment of NSCLC recommend the use of cisplatin-containing combinations because these have been shown to improve survival in comparison with the best supportive treatments (NSCLCEP, 1997). However, the toxic effects of these combinations (particularly on the kidneys, which require greater hydration) contraindicate their use in both nephropathic and cardiopathic patients. Severe and prolonged cisplatin-induced nausea and/or vomiting is another major limitation that can only be partially controlled by modern anti-emetics.

Some studies have shown that carboplatin has the same activity and is better tolerated (Klastersky et al, 1990), but at the cost of greater haematological toxicity. Other more recent studies have shown that taxanes are also active against NSCLC (especially when associated with platinum derivatives) and may lead to even 50% objective response rates (Langer et al, 1995; Bonomi, 1999). However, as in the case of those based on cisplatin, the toxicity of these combinations limits their use to patients with good or excellent general health.

A phase I study of the combination of cisplatin, gemcitabine and vinorelbine found an objective response rate in 51% of NSCLC patients, but 26% experienced grade 4 neutropenia (Frasci et al, 1997).

Our study was specifically designed to evaluate the feasibility and activity of gemcitabine plus vinorelbine in patients whose age, concomitant diseases or general physical condition normally excludes the use of aggressive treatment programmes. The response rate was better than that reported when the two drugs are administered separately, and similar to that obtained using the first-generation cisplatin combination. It is also worth noting that the 1-year actuarial survival rate is 31.1% and that the median survival of the responsive patients has not been reached after 20 months. However, it must be pointed out that ten of the responding patients also received radiotherapy and so not all of the survival benefit can be attributed to the chemotherapy.

The fact that 10% of the patients in stage IIIb were able to undergo surgery despite their age or poor condition suggests that the combination could also be tested as a neo-adjuvant treatment.

The tolerability of the treatment is further underlined by the fact that there was almost complete concordance between the planned treatment schedule and the actually administered doses.

**Table 3** Phase II studies of gemcitabine (GEM) plus vinorelbine (NVB)

| Author | Phase | NVB – GEM (mg) | Timing (day) | Patients (*n*) | OR* (%) |
|---|---|---|---|---|---|
| Esteban 1998 | I–II | 30–1250 | 1,8 | 32 | 43.3 |
| Cigolari 1998 | I–II | 25–1000 | 1,8 | 25 | 36 |
| | | 25–1200 | | 25 | 20 |
| Lilenbaum 1998 | II | 25–1250 | 1,8 | 13 | 27 |
| Feliu 1998 | II | 25–1000 | 1,8,15 | 36 | 22.5 |
| De Candis 1998 | II | 25–1250 | 1,8 | 32 | 34 |
| Isokangas 1999 | I–II | 30–1000 | 1,8,15 | | |
| | | | | 45 | 40 |
| | | 35–1200 | 1,15 | | |
| Lorusso 2000 | I–II | 30–1200 | 1,8 | 52 | 36 |

*OR = objective response

The doses were selected on the basis of their feasibility and optimal tolerability. In particular, the decision not to administer the treatment on day 15 made it possible to reduce haematological toxicity to a significant extent. Although only two full papers (Isokangeas et al, 1999; Lortusso et al, 2000) have so far been published concerning this treatment regimen, a number of abstracts have been submitted to international congresses describing the use of these two drugs in phase I or II trials (see Table 3). In these studies, the results obtained using various doses were no better than ours, whereas a similar treatment regimen administered up to day 15 led to a toxic death rate of 5%(Feliu et al, 1998).

In conclusion, despite the fact that the 1-year survival is similar to that achieved with vinorelbine alone (LeChevallier, 1996) and that there is therefore still a need to compare the results obtained using the combination with those obtained using the single agent, it can be said that the administration of gemcitabine plus vinorelbine is effective and well tolerated by NSCLC patients, and that its excellent safety profile encourages its use in elderly and unfit patients.

### ACKNOWLEDGEMENT

We would like to thank the Associazione Oncologica Bergamasca for supporting this study.

*© 2000 Cancer Research Campaign*

### REFERENCES

Bonomi P (1999) Review of paclitaxel/carboplatin in advanced non-small cell lung cancer. *Semin Oncol* **26**(suppl 2): 55–59

Bunn PA Jr (1993) Future directions in the management of non-small cell lung cancer. *Lung Cancer* **9**(suppl 2): 91–127

Cigolari S, Gridelli C, Frontini L, Gulisano M, Robbiati SF, Farris A, Clerici M, Castiglione F, Piazza E, Ianniello GP and Perrone F (1998) Gemcitabine + vinorelbine (GEMVIN) in the treatment of advanced non-small cell lung cancer (NSCLC). Sequential phase I and phase II trials. *Proc ESMO* **9**: 87 (abs 419P)

De Candis D, Bidoli P, Cresta S, Artale S, Masi G and Bajetta E (1998) Phase II study of gemcitabine (GEM) plus navelbine (NVB) in previously untreated stage III or IV non-small cell lung cancer (NSCLC). *Proc ESMO* **9**: (abs 460) 95

Esteban E, Llano JLG, Vieitez Jm, Fra J, Puertas J, Estrada E, Palacio I, Mumiz I, Buesa JM and Lacave AJ (1998) Phase I/II study of gemcitabine plus vinorelbine in non-small cell lung cancer (NSCLC). *Proc Am Soc Clin Oncol* **17**: (abs 1855) 482a

Feliu J, Lopez-Gomez L, Madronal C, Jalon I, Garcia-Giron J, Castro J, Martinez B, Iglesias J, Zamora P and Gonzales-Baron M (1998) Phase II study of gemcitabine-vinorelbine (G-V) in patients with advanced non-small cell lung cancer (NSCLC) over 70 year-old or with contraindication to receive cisplatin. *Proc ESMO* **9**: (abs 411P) 85

Fossella FV, Lee JS, Shin DM, Calayag M, Huber M, Perez-Soler R, Murphy WK, Lippman S, Benner S and Glisson B (1995) Phase II study of docetaxel for advanced or metastatic platinum-refractary non-small cell lung cancer. *J Clin Oncol* **13**: 645–651

Frasci G, Panza N, Comella P, Nicolella GP, Natale M, Pacilio C, Gravina A, Caputi V, Botti G and Comella G (1997) Cisplatin, gemcitabine and vinorelbine in locally advanced or metastatic non-small-cell lung cancer: a Phase I study. *Ann Oncol* **8**: 1045–1048

Fukuoka M, Niitani H, Suzuki A, Motomiya M, Hasegawa K, Nishiwaki Y, Kuriyama T, Ariyoshi Y, Negoro S and Masuda N (1992) A phase II study of CPT-11, a new derivative of camptothecin, for previously untreated non-small cell lung cancer. *J Clin Oncol* **10**: 16–20

Gatzemeier U, Heckmayr M, Neuhauss R, Schluter I, Pawel JV, Wagner H and Dreps A (1995) Phase II study with paclitaxel for the treatment of advanced inoperable non-small cell lung cancer. *Lung Cancer* **12**:(suppl 2) 101–106

Goss GD, Logan DM, Newmann TE and Evans WK (1997) Use of vinorelbine in non-small cell lung cancer. Provincial Lung Disease Site Group. *Cancer Prev Control* **1**: 28–38

Isokangas OP, Knuuttila A, Halme M, Mantyla M, Lindstrom I, Nakkanen V, Viren M, Joensuu H and Mattson K (1999) Phase II study of vinorelbine and gemcitabine for inoperable stage IIIB-IV non-small-cell lung cancer. *Ann Oncol* **10**: 1059–1063

Klastersky J, Sculier JP, Lacroix H, Dabouis G, Bureau G, Libert P, Richez M, Ravez P, Vandermoten G and Thiriaux J (1990) A randomized study comparing cisplatin or carboplatin with etoposide in patients with advanced non-small cell lung cancer: European Organization for Research and Treatment of Cancer protocol 07861. *J Clin Oncol* **8**: 1556–1562

Langer CJ, Leigthon JC, Comis RL, O'Dwyer PJ, McAleer CA, Bonjo CA, Engstrom PF, Litwin S and Ozols RF (1995) Paclitaxel and carboplatin in combination in the treatment of advanced non-small cell lung cancer: A phase II toxicity, response, and survival analysis. *J Clin Oncol* **13**: 1860–1870

Le Chevalier T (1996) Chemotherapy of non-small-lung cancers. *Presse Med* **25**: 1699–1703

Lilenbaum RC, Green MR (1993) Novel chemotherapeutic agents in the treatment of non-small cell lung cancer. *J Clin Oncol* **11**: 1391–1402

Lilenbaum RC, Schwartz MA, Cano R, Krill E, Lutzky J, Baulstein A, Seigel L, Viales M, Dominguez CJ and Davila E (1998) Gemcitabine (GEM) and navelbine (NVB) in advanced non-small cell lung cancer (NSCLC). *Proc Am Soc Clin Oncol* **17**:494a (abs 1901)

Lorusso V, Carpagnano F, Frasci G, Panza N, Di Rienzo G, Cisternino ML, Napoli G, Orlando S, Cinieri S, Brunetti C, Palazzo S and De Lena M (2000) Phase I/II study of gemcitabine plus vinorelbine as first-line chemotherapy of non-small cell lung cancer. *J Clin Oncol* **18**: 405–411

Noble S and Goa KL (1997) Gemcitabine. A review of its pharmacology and clinical potential in non-small cell lung cancer and pancreatic cancer. *Drugs* **54**: 447–472

Non-Small Cell Lung Cancer Collaborative Group (1995) Chemotherapy in non-small cell lung cancer: a meta-analysis using updated data on individual patients from 52 randomized clinical trials. *BMJ* **311**: 899–909

Non Small Cell Lung Cancer Expert Panel (1997) Clinical practice guidelines for the treatment of unresectable non-small cell lung cancer. *J Clin Oncol* **15**: 2996–3018

Parker SL, Tong T, Bolden S and Wingo PA (1996) Cancer statistics. *CA Cancer J Clin* **65**: 5–27

Simon R (1989) Optimal two-stage design for phase II clinical trials. *Control Clin Trials* **10**: 1–10

Souquet PJ, Chauvin F, Boissel JP, Cellerino R, Cormier Y, Ganz PA, Kaasa S, Pater JL, Quoix E and Rapp E (1993) Polychemotherapy in advanced non-small cell lung cancer: A meta-analysis. *Lancet* **342**: 19–21

The Elderly Lung Cancer Vinorelbine Italian Study Group (ELCVIS) (1999) Effects of vinorelbine on quality of life and survival of elderly patients with advanced non-small cell lung cancer. *J Natl Cancer Inst* **91**: 66–72

Zanetti R, Buiatti E, Federico M and Micheli A (1998) *Fatti e cifre dei tumori in Italia*. II Pensiero Scientifico: Roma

 *© 2000 Cancer Research Campaign*
